# Huntington's disease biomarker progression profile identified by transcriptome sequencing in peripheral blood

**DOI:** 10.1038/ejhg.2014.281

**Published:** 2015-01-28

**Authors:** Anastasios Mastrokolias, Yavuz Ariyurek, Jelle J Goeman, Erik van Duijn, Raymund AC Roos, Roos C van der Mast, GertJan B van Ommen, Johan T den Dunnen, Peter AC 't Hoen, Willeke MC van Roon-Mom

**Affiliations:** 1 Department of Human Genetics, Leiden University Medical Center, Leiden, The Netherlands; 2 Leiden Genome Technology Center, Leiden University Medical Center, Leiden, The Netherlands; 3 Department of Medical Statistics and Bioinformatics, Leiden University Medical Center, Leiden, The Netherlands; 4 Department for Health Evidence, Radboud University Medical Center, Nijmegen, The Netherlands; 5 Department of Psychiatry, Leiden University Medical Center, Leiden, The Netherlands; 6 Center for Mental Health Care Delfland, Jorisweg 2, Delft, The Netherlands; 7 Department of Neurology, Leiden University Medical Centre, RC, Leiden, The Netherlands

## Abstract

With several therapeutic approaches in development for Huntington's disease, there is a need for easily accessible biomarkers to monitor disease progression and therapy response. We performed next-generation sequencing-based transcriptome analysis of total RNA from peripheral blood of 91 mutation carriers (27 presymptomatic and, 64 symptomatic) and 33 controls. Transcriptome analysis by DeepSAGE identified 167 genes significantly associated with clinical total motor score in Huntington's disease patients. Relative to previous studies, this yielded novel genes and confirmed previously identified genes, such as *H2AFY,* an overlap in results that has proven difficult in the past. Pathway analysis showed enrichment of genes of the immune system and target genes of miRNAs, which are downregulated in Huntington's disease models. Using a highly parallelized microfluidics array chip (Fluidigm), we validated 12 of the top 20 significant genes in our discovery cohort and 7 in a second independent cohort. The five genes (*PROK2, ZNF238, AQP9, CYSTM1 and ANXA3*) that were validated independently in both cohorts present a candidate biomarker panel for stage determination and therapeutic readout in Huntington's disease. Finally we suggest a first empiric formula predicting total motor score from the expression levels of our biomarker panel. Our data support the view that peripheral blood is a useful source to identify biomarkers for Huntington's disease and monitor disease progression in future clinical trials.

## Introduction

Huntington's disease (HD) is a heritable neurodegenerative disorder that manifests itself through cognitive, psychiatric and motor symptoms. The pathology is caused by an expanded CAG repeat in the *HTT* gene, resulting in a mutant huntingtin protein. Patients also develop peripheral pathology^1^ and increasing evidence indicates that peripheral inflammation has a role as a disease progression modulator.^2^ HD brain tissue is characterized by mutant protein aggregate formation and neuronal cell loss, with transcriptional deregulation as a prominent feature.^3, 4^ Several mechanisms have been implicated in this deregulation such as histone modifications, transcription factor impairment and aberrant miRNA expression.^5^ For HD clinical trials, it is important to identify disease progression biomarkers. Longitudinal studies have shown that imaging biomarkers and clinical measures provide valuable information.^6^ However, clinical measures can be subject to inter-rater variability and imaging is expensive. A biomarker should be able to identify changes before clinical symptoms, should be easily obtained and should respond well to disease-modifying interventions. As it is impossible to measure molecular biomarkers in the brain, the use of more accessible tissues has been proposed, such as blood. Leukocytes involved in immune system regulation make blood an ideal source for identifying HD events such as peripheral inflammation. In addition, as huntingtin is ubiquitously expressed, mutant huntingtin-specific changes could also be reflected by gene expression changes in blood. Several studies have identified HD blood mRNA changes using microarray technology, but it has proven difficult to validate these across studies.^7, 8, 9^

Advances in next-generation sequencing offer new inroads to study the transcriptome. The digital nature of next-generation sequencing allows for accurate quantification of unknown transcripts, low- and high-abundance transcripts. Sequence-based methods allow the measurement of known as well as unknown transcripts, thus obviating the past limitation to the microarray content. In addition, sequence-based methods are more precise than microarrays and more robust across experiments because of much greater depth and the absence of the background signal and cross hybridization issues that were associated with microarrays.^10^ One such method, the 3′ digital expression profiling (DGE/DeepSAGE) creates 21 base pair sequences (tags) near the 3′ ends of polyadenylated mRNAs^11^ and uniquely identifies transcripts using these tags. Thus, by counting the matching transcripts one can estimate differences in gene expression between samples across a large dynamic range. In comparison with full-length RNA sequencing, DeepSAGE has the advantage of comprehensive coverage of all (transcribed) genes at great depth, at the cost of not detecting different splice variants. In this study, we investigated the suitability of blood to identify HD transcriptomic biomarkers, validated the outcome in an independent cohort and derived a first empiric panel of biomarkers capable of predicting HD motor scores. Finally, we examined whether patient gene expression profiles could provide information about HD-affected biological pathways.

## Materials and methods

### Cohort assessment and characteristics

Peripheral blood from 33 controls, 27 presymptomatic mutation carriers and 64 symptomatic mutation carriers were collected for the discovery cohort and independent validation cohort from 12 symptomatic mutation carriers and 11 controls. Collection was done with IRB approval and after informed consent. All subjects were examined by an experienced neurologist using the motor section of the Unified Huntington's Disease Rating Scale (UHDRS) as described previously.^12^ All the controls were free of known medical conditions. Age considered for the analysis was the age at the time of blood collection. For a detailed summary of the study cohort's average age, gender composition, UHDRS TMS and HD progression total functional capacity scores (TFC) see Supplementary Table S3.

### RNA isolation and DeepSAGE library production

RNA isolation and cDNA library production were performed as described previously.^13^ In short total RNA was extracted from PAX gene blood tubes (Qiagen, Venlo, The Netherlands), and 1 *μ*g of total RNA was used to synthesize double-stranded cDNA constructs for next-generation sequencing.

### Sequence processing

Illumina GA Pipeline (version 1.5.1) was used for data sequence processing. The FASTQ files were analyzed using the open source GAPSS_B pipeline (http://www.lgtc.nl/GAPSS) as described previously.^13^ In addition, a custom Perl script was used to obtain gene annotations from Biomart, and a custom python script was used to count the tags in each Ensembl gene using the sam output files from bowtie. To avoid batch effects, the samples were randomized during RNA isolation and DeepSAGE sample preparation. To identify potential sample swaps and contaminations, all samples were checked for the correct expression of *XIST* and *RPS4Y1* gender-specific genes. Batch effects were assessed using multidimensional scaling (MDS) plots for gender, sequencing flow cell and disease stage and by using the edgeR bioconductor package for RNA-Seq. The sequencing gene expression data used for this study have been deposited in the Gene Expression Omnibus (GEO) database under accession number GSE51799.

### Fluidigm RT-qPCR

cDNA synthesis was performed using 1 *μ*g of total RNA from each blood sample and using random hexamer primers with the Transcriptor First Strand cDNA synthesis kit (Roche, Basel, Switzerland). cDNA was diluted four times and 1.25 *μ*l of each sample was preamplified using 2.5 *μ*l of 2x Taqman pre-amplification master mix (Applied Biosystems, Waltham, MA, USA) and 1.25 *μ*l of the primer pool (0.2 pmol each primer/*μ*l). The preamplifications were performed using a 10 min 95 °C denaturation step and 14 cycles of 15 s at 95 °C and 4 min at 60 °C. The preamplified reactions were diluted 5 × times in H_2_0. Five microliters from a sample mix containing preamplified cDNA and amplification Master mix (20 mM Mgcl2, 10 mM dNTPs, FastStart Taq polymerase, DNA binding Dye loading reagent, 50 × RO ×, 20 × Evagreen) was loaded into each sample inlet of the 48.48 dynamic array chip (Fluidigm Corporation, San Francisco) and 5 *μ*l from an assay mix containing DA assay loading reagent, as well as forward and reverse primers (10 pmol/*μ*l), was loaded into each detector inlet. The chip was then placed on the NanoFlexTM 4-IFC Controller for loading and mixing. After loading, the chip was placed on the BioMarkTM Real-Time PCR System using a cycling program of 10 min at 95 °C followed by 40 cycles of 95 °C for 15 s and 60 °C for 30 s and 72 °C for 30 s. Data were analyzed using the BioMark Gene Expression Data Analysis software to obtain Ct values and/or ΔCt values. Fluidigm data were corrected for differences in input RNA using the geometric mean of three reference genes *ACTB*, *HPRT* and *RPL22*. The array accommodated reactions for all 48 validation samples and 23 genes in duplicate (duplicate values were averaged).

### Statistical analysis

All DeepSAGE downstream analyses were performed at the gene level, and in case of multiple SAGE tags per gene, for example, as a consequence of alternative polyadenylation, tags were summarized. All the tag counts for a certain gene across all 124 samples were summarized. Low-abundance genes with <124 tags were removed as were the top three overabundant genes (*HBA1*, *HBA2* and *HBB*). Gene expression analysis was performed using the limma package and the voom function for RNA-seq data and by applying linear modeling and empirical Bayes statistics.^14^ The model tested gene expression as a function of the subject's total motor score (TMS), while accounting for gender, age and relative cell content (measured by the ratio of hemoglobin tags *versus* total aligned tags per sample) as confounders. Fluidigm expression analysis was performed using the linear modeling function in R and by testing the individual Δct expression values against the subject's TMS, while accounting for gender and age. Global test pathway analysis was performed using the same model as was used for the DeepSAGE analysis. For GO pathway analysis, the top *P-*value pathways that consisted of a minimum of 10 genes were reported. For IPA analysis, the top 250 DeepSAGE genes were used (*P-*value <0.001). For TMS prediction a linear regression model with a lasso penalty was fitted using the R package penalized, optimizing the lasso tuning parameter using leave-one-out cross-validation.^15^ The effects of age and gender were not penalized.

## Results

### Gene expression analysis

**Figure 1:**
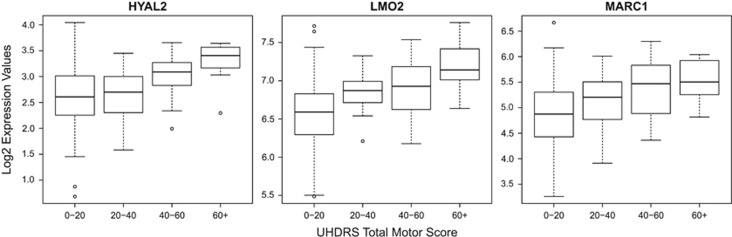
Boxplots of the DeepSAGE expression values for the top three upregulated genes discovered from linear modeling with TMS and for all 124 samples. The plot confirmed our linear modelling analysis and demonstrated a gradual increase in gene expression across the different total motor score groups.

Samples were sequenced at an average library size of 23.5 million tags. Alignment to the human genome resulted in an average library size of 20.4 million tags with at least one reported alignment (87.1%). A detailed description of the sequenced samples RNA integrity numbers (RIN) and sequence alignment characteristics can be seen in Supplementary Table S4. After removal of very low abundance genes, we could reliably detect a total of 16 657 genes. To find HD-specific stage or progression biomarkers, the DeepSAGE gene expression data were modeled as a function of the individual UHDRS total motor score (TMS), while accounting for gender, age and the percentage of hemoglobin tags (a proxy for the reticulocyte content) as confounders. The TRACK HD study has shown that in presymptomatic HD gene carriers the motor score scale (0–124) is a strong predictor of subsequent clinical conversion.^6^ Our HD group consisted 27 presymptomatic (TMS=2.4±1.8) and 64 symptomatic (TMS=37.4±24.3). After linear modeling, a total of 167 genes significantly associated with motor score at an adjusted *P-*value of 0.05 or less, suggesting that these constitute potential disease stage biomarkers. Of these 167 genes, 99 were positively associated with motor score and upregulated in HD samples compared with controls, whereas 68 were negatively associated and downregulated. The top 10 upregulated and top 10 downregulated genes are shown in Table 1. When we grouped the samples based on TMS, we could confirm our linear modeling results. Boxplots for the top three upregulated genes showed a gradual increase in gene expression with increasing TMS (Figure 1). A full list of all the genes significantly associated with TMS as well as with total functional capacity score (TFC) disease staging is provided in Supplementary Tables S5 and S6, respectively. Reassuringly in the TFC-based analysis, 60% of the genes were the same as the TMS-based significant genes. Among the top TMS *P*-value significant genes were genes involved in the regulation of circadian rhythm such as prokineticin 2 (*PROK2*), genes associated with motor learning such as protein tyrosine phosphatase non receptor 4 (*PTPN4*) and genes implicated in the development of the brain cortex such as G protein-coupled receptor 56 (*GPR56*).^16, 17, 18^ The genes with the biggest expression change but lacking statistical significance were the small nuclear RNA host gene 9 (*SNGH9*) and the major histocompatibility complex class II DQ alpha1 gene (*HLA-DQA1*). *HLA-DQA1* has been previously reported as a candidate RNA biomarker in human lymphocyte microarray data from HD patients, ranking among the top most changed genes.^9^ The highest expressed significant gene was S100 calcium binding protein A9 (*S100A9*) with a log_2_ expression value of 11.7, while the lowest expressed significant gene was sperm acrosome associated 3 (*SPACA3*) with a value of −2.8, indicative of the high dynamic range of the sequencing platform (2^11.7−(−2.8)^=23 170 fold).

Using EBI Gene Expression Atlas (http://www.ebi.ac.uk/gxa/) and literature searches, we found that 40 of the 167 genes had been previously reported as differentially expressed in at least one HD gene expression study with the same direction in expression change. These included mechanistic target of rapamycin (*MTOR*), a potential target for therapy in HD, H2A histone family member Y (*H2AFY*), a gene whose transcript levels have been recently reported to mark HD activity in human and mouse, CDC-like kinase 3 (*CLK3*) another gene from the top 99 genes from the previous study, and aquaporin 9 (*AQP9*), a gene that has been described as a potential biomarker in blood.^8, 9, 19^

### Global test pathway analysis

To elucidate affected biological pathways in HD blood that were associated with TMS, we used the Global test bioconductor package.^20^ We included KEGG pathways, GO terms and predicted/validated target genes of miRNAs (BROAD-GSEA). In the KEGG pathway analysis (see Supplementary Table S7), we found terms frequently reported in HD and neurodegenerative disorder pathway analyses such as neuroactive ligand receptor interaction, amyotrophic lateral sclerosis and long-term depression. We also found less common terms such as the pentose phosphate pathway (PPP), Jak-STAT signaling and type II diabetes mellitus. The genes that contributed most to PPP were glucose phosphate isomerase (*GPI*), aldolase A (*ALDOA*), phosphogluconolactonase (*PGLS*) and transketolase-like 1 (*TKTL1*), an enzyme linking PPP with the glycolytic pathway. Mitochondria-associated metabolic dysfunction and increased glycolytic rate have been previously associated with HD.^21^ The Jak-STAT pathway, a common signaling pathway used by many cytokines, was characterized by the upregulation of serine-threonine protein kinase (*AKT1*), suppressor of cytokine signaling 3 (*SOCS3*), son of sevenless homolog 2 (*SOS2*) and interferon-alpha/beta receptor beta chain (*IFNAR2*). Finally, for diabetes for which an increased frequency in HD patients has been previously described, the most significant genes were *MTOR* and protein kinase C delta (*PRKCD*).^22^ In the GO analysis, we identified terms such as NADP binding, positive regulation of interleukin 6 production and response to cholesterol. The most significant genes for NADP binding were neuronal nitric oxide synthase 1 (*NOS1*), flavin containing monooxygenase 4 (*FMO4*) and homocysteine methyltransferase reductase (*MTRR*). The deregulation of genes linked to response to cholesterol could also be important as cholesterol biosynthesis has been shown to be impaired in HD cells, while Leoni *et al.*^23^ have demonstrated that 24OHC, a brain cholesterol turnover marker, correlated with disease progression. All the genes reported for response to cholesterol can be seen in a Global test covariate plot in Supplementary Figure S5A. This result was also in agreement with Chou *et al.*^24^ who showed that the mutant HTT protein suppresses the secretion of CCL5. The analysis for enrichment of target genes of miRNAs showed enrichment of miR-138 and miR-218 targets. These miRNAs were found downregulated in YAC128 and R6/2 HD mouse models.^25^ For the miR-138 and miR-218 target genes, a separate enrichment analysis, using DAVID (http://david.abcc.ncifcrf.gov), showed that terms enriched specifically for miR-138 target genes were histone modification and axon guidance, while terms enriched specifically for miR-218 target genes were ubiquitin-like conjugation, proto-oncogene and mental retardation. Other potentially interesting miRNAs that were identified previously were miR-18a, miR-504, miR-337 and miR-492.^26, 27^ To further validate our Global test pathway analysis results and obtain a better visual representation of the interconnections of the genes involved in the above biological processes, we also analyzed our data through the use of the Ingenuity Pathway Analysis (IPA) (Ingenuity Systems, www.ingenuity.com). Top diseases and functions reported by IPA network analysis were nervous system development, skeletal and muscular disorders but also immune cell trafficking and inflammatory response (Supplementary Table S8). The gene network plot for the genes and molecules involved in the IPA network 6 and for skeletal and muscular disorders, connective tissue disorder and cancer is shown in Supplementary Figure S5B. Interestingly, this gene plot interconnected terms such as histones, 26s proteasome, pro-inflammatory cytokines, Hsp70 and insulin; all of which have previously been implicated in HD. Canonical pathway analysis using IPA further confirmed our initial Global test results, as common pathways reported were those of diabetes mellitus, Toll-like receptor and T-cell receptor signaling. Finally, upstream regulators from our top genes were reported to be IL-2, IL-6 and IL-12(complex) by IPA analysis, which was also in good correlation with the Global test analysis.

### Validation

**Figure 2:**
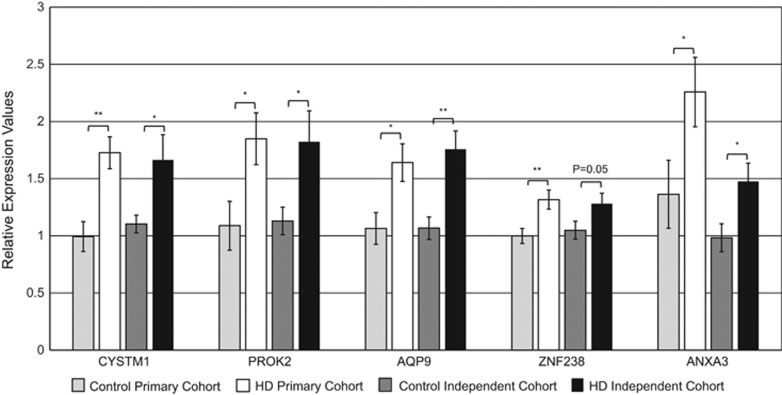
Relative expression of the most significant Fluidigm RT-qPCR genes across the two independent cohorts for controls and HD patients. Asterisks represent statistical significance from a Student's *t*-test (*P<0.05, **P<0.01). Error bars represent SEM values.

To validate the DeepSAGE gene expression results, we performed nanoliter RT-qPCR using the Fluidigm Biomark microfluidics chip^28^ using 25 samples from the original discovery cohort as technical validation, supplemented with 23 patient and control samples as a biological validation in an independent cohort. Twenty genes in total, all from our DeepSAGE list of 167 significantly differentially expressed genes, were examined; the top 12 based on *P*-value, 6 further down the 167 gene list based on differential expression in previous HD studies (*H2AFY, AQP9, ANXA3, RGS14, ZNF238, NOL3*) and another 2 genes from the same list based on possible involvement in HD pathology (*CEBPA, TAF15*).^3, 8, 9, 19, 29, 30, 31^ Fluidigm data were analyzed using a linear model as a function of TMS, while accounting for gender and age. In the basic validation cohort, 12 out of the 20 genes tested were significantly associated with TMS, while, in the independent validation cohort, 7 out of the 20 genes were significant (see Table 2). Most other genes, while not reaching significance, showed trends in the same direction as in the discovery cohort. Five of the 20 genes (*PROK2*, *ZNF238*, *AQP9*, *CYSTM1* and *ANXA3*) were the most robust and significantly associated with TMS in both the discovery and the independent cohort. The intergroup relative expression levels of these five genes across HD *versus* control samples, irrespective of TMS, can also be seen in Figure 2. Finally, when the linear modeling analysis was performed on all Fluidigm samples (*n*=48), we were able to validate 12 of the 20 genes tested (see rightmost column of Table 2).

### Biomarker motor score prediction

**Figure 3:**
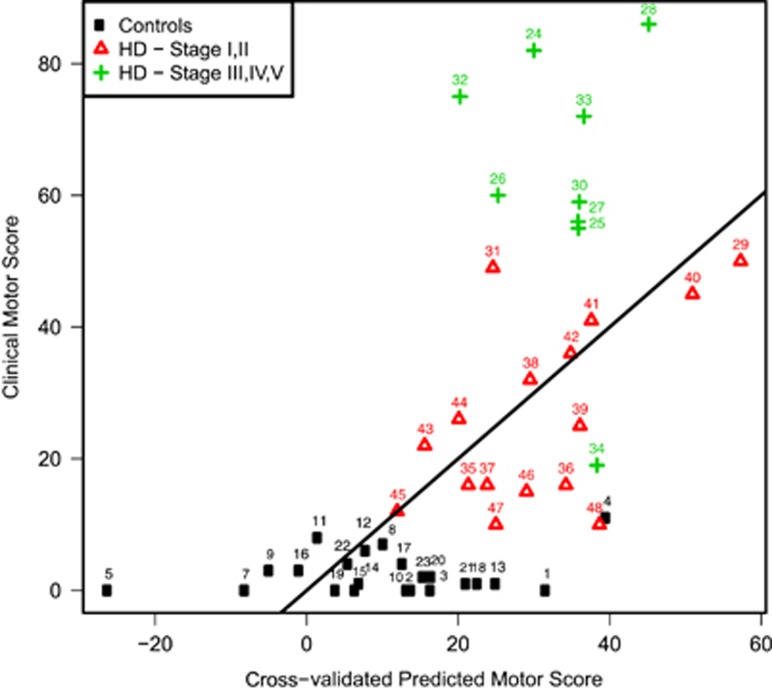
Plot of clinical TMS against cross-validated predicted TMS based on Fluidigm RT-qPCR gene expression data. The cross-validated motor score is predicted for each subject by a model trained on a data set in which the subject itself was left out. Stage classification was based on total functional capacity (TFC) scores (Stage I, II=TFC score 7–13/Stage III-V=TFC score 0–7).

**Figure 4:**
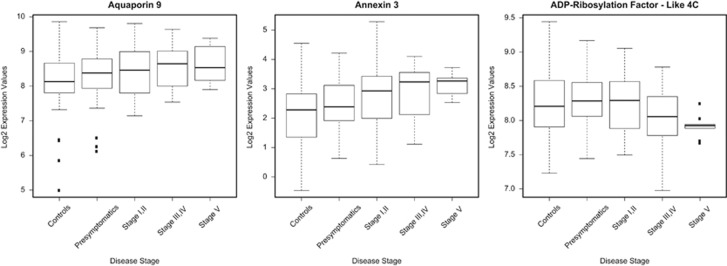
DeepSAGE gene expression levels for the best three TMS predictor genes as these were reported from the LASSO algorithm prediction analysis and across controls, presymptomatics and different HD symptomatic stages.

To evaluate which panel of genes would optimally predict TMS, we fitted a linear regression model with a lasso penalty using the Fluidigm expression data, age and gender as predictors and TMS as the response. The gene expression values of three genes *(AQP9*, *ANXA3* and *ARL4C*), together with age and gender, were the best predictors of TMS. The last gene (*ARL4C*) was non-significantly downregulated in HD blood and specifically served the purpose of enlarging the ‘biomarker chip' set towards tolerance for smaller individual gene changes, providing additional informativeness. The results of the cross-validated prediction analysis can be seen in Figure 3. The prediction model performed better for earlier disease stages (Stage I, II), while it was less accurate for later stages (Stage III-V) and especially for patients with a motor score of 50 points and over. Only one patient was assigned a predicted TMS >50 points (patient no.29). This patient was the oldest HD carrier (>70 years). We also observed that for one patient the blood-based signature indicated a higher predicted motor score compared with the clinical motor score. This could be explained by the fact that this patient had a much lower TFC score (TFC=4) compared with other patients with similar motor score, indicative of a more advanced disease stage. Finally, the control sample with the highest clinical motor score (control no.4) was our oldest control sample (69 years) and also received a higher predicted score. When we plotted the DeepSAGE gene expression levels of these three genes across the controls, the presymptomatic carriers and the different HD TFC-based disease stages, we could confirm that for *ANXA3* and *AQP9* there was an increase in gene expression even in the presymptomatic stage. For *ARL4C*, contrary to *ANXA3* and *AQP9* there was a decrease in gene expression, the expression changes were more prominent in the more advanced disease stages and hence provided complementary information to the other two genes (Figure 4). On the basis of this analysis, we formulated the following TMS predictive equation to measure the disease stage based on gene expression of the three genes:


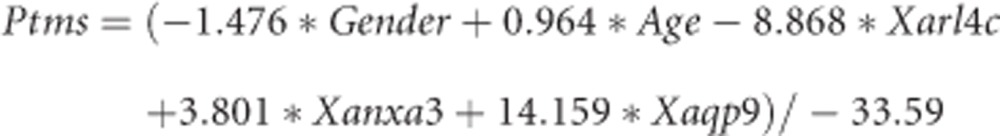


where *Ptms* is the gene expression predicted TMS, gender is 0 or 1 for male or female and *X* the respective, housekeeping gene corrected*, ΔCt* RT-qPCR value.

## Discussion

To date, thousands of disease biomarkers have been published while <100 have been validated in independent cohorts.^32^ This inability to validate disease biomarkers has been attributed to the lack of large enough study cohorts as well as standardization in sample collection and storage.^33^ For HD, validation has been even more challenging as the disease presents itself through a variety of symptoms and progression rates. For these reasons, we performed gene expression profiling, taking advantage of the sensitivity of next-generation sequencing and Fluidigm technologies, and our experience in standardized blood collection and sample analysis.^34, 35^ Using the UHDRS TMS as a clinical parameter, we identified a set of 167 genes differentially expressed in HD blood. Furthermore, we validated our findings by a targeted approach, using an entirely different technology. Technical validation (in the same cohort) confirmed 12/20 of the discovered genes and biological validation (in a different cohort) confirmed 7/20 of the discovered genes in a different cohort. Our discovery and validation cohorts (*n*=124 and *n*=48) are to our knowledge among the largest to have been used in HD gene expression studies. In contrast to previous studies, we have selected a sizable group of 20 genes for validation in duplicate (~2300 reactions). Indeed, the very fact that so many of the top 20 discovered genes can be validated argues in favor of the robustness of the discovery approach. Genes with more variation or smaller changes in principle are more difficult to validate in a small cohort. Yet, we should stress that these biomarkers presently constitute a candidate biomarker set that requires further validation in other HD cohorts before further used in a clinical setting.

The Fluidigm qPCR analysis yielded a panel of five genes (*PROK2, ZNF238*, *AQP9, CYSTM1* and *ANXA3*) as a potential HD biomarker set, and this was validated in both the original cohort and an independent validation cohort. *PROK2* is expressed in the suprachiasmatic nucleus (SCN) and has been proposed to have a role in the regulation of circadian rhythms.^17^ Circadian rhythm alterations have been shown to correlate with cognitive impairment in HD^36^ and in HD models pharmacological imposition of sleep slows cognitive decline and reverses deregulation of *PROK2*.^37^ As a blood marker of HD progression PROK2 is very promising, since this could also be reflecting brain changes. *ZNF238* is a transcriptional repressor involved in brain development and myogenesis,^38^ and increasing evidence suggests that gene repression mechanisms are associated with HD.^39, 40^ This is in agreement with the reported involvement in HD of *H2AFY*, which is also involved in transcriptional repression, and further studies link HD with *SP1*, another zinc-finger protein.^41^ Aquaporins are water selective channels with possible roles in the nervous system and expression levels were upregulated after brain injury.^42^ The presence of *AQP9* in blood could represent peripheral or central inflammatory events, as a recent gene expression study showed that the mRNA levels of *AQP9* and four other genes can discriminate patients with chronic inflammation from controls.^43^ *CYSTM1* is a relatively unknown gene and bioinformatics analysis has demonstrated a role in stress response and confer tolerance to heavy metals such as cadmium and copper.^44^ *ANXA3* was upregulated in two neuronal injury models.^45, 46^ It is important to note that the levels of annexin *ANXA1* have also been found upregulated in a previous gene expression study in HD blood.^8^

Our pathway analysis showed a wide range of processes changed in HD. The most prominent terms pointed towards the involvement of the immune system. It has been suggested by previous studies that pro-inflammatory cytokines such as IL-6, IL-8 and TNF-*α* can be used as peripheral HD biomarkers.^47, 48^ Other terms such as diabetes mellitus could also be interesting as mouse models of HD can develop diabetes mellitus,^49^ and it was shown that type II diabetes exhibits common features with other neurodegenerative disorders.^50^ Finally, we discovered enrichment of target genes of miRNAS (miR-138/218) previously reported to be downregulated in HD models. This warrants further investigation as miR-9 was found to be downregulated in human HD brain samples and target complexes, such as *REST,* that regulates neuronal gene expression in non-neuronal tissues.^51^ A disadvantage of whole blood may be considered its cellular heterogeneity. The more informative white blood cells comprise a small percentage of the total cell population, while 95% of blood consists of red blood cells, with hemoglobin transcript percentages, ranging from 30 to 90%. This could well account for the fact that until now less-sensitive techniques failed to replicate results between different HD blood microarray studies.^52^ For the same reason, in the past, most expression studies used isolated peripheral blood mononuclear cells. However, it is not always possible to process samples directly after collection and preparation delays have been shown to induce biases.^53^ In the present study, taking advantage of the digital nature of sequencing, we identified differentially expressed genes across a wide dynamic range, with high sensitivity, directly from whole blood. This provides a clearer image of the transcriptional alterations in HD, although biomarkers with higher expression will be more useful and easier to detect with less-sensitive routine techniques. Our motor score prediction analysis showed that the gene expression predictive power was stronger for early-stage and weaker for later-stage patients. While this could be explained by the increasing impact of generalized tissue degeneration in late disease stages, the increased reliability in earlier stages is in fact of major benefit, as, notably in this early phase, robust therapeutic read-outs are challenging. Furthermore, previous gene expression studies have found small individual gene expression changes in HD blood. In the future and for a potential ‘biomarker chip' to survive further validation, a larger group of genes may be required that will better allow for variation in individual gene expression changes. For this reason, we used the predictive capabilities of the LASSO algorithm to see which genes would jointly perform most optimally in UHDRS TMS prediction. The formula we have derived links a small set of easily definable gene expression levels to the UHRDS Total Motor Score, and is thus a promising candidate biomarker set to monitor disease state, progression and putative therapeutic effect of interventions. Taking into account the great symptomatic variability in HD patients, different sets of biomarkers can be further trained and optimized, depending on the disease stage that is most prominent in the group of patients included in each study.

Considering the complexity of HD most likely a collection of biomarkers will best define disease progression and response to therapy. The biomarker changes found in this study monitor disease progression in blood and may be relatively independent of the changes taking place in the brain. Such biomarkers, if validated clinically, may be useful as surrogate markers to test the effectiveness of therapeutic strategies even when they may not have a robust relationship with actual clinical end points.^54^

Owing to the design of our study, comparing various HD stages with unaffected controls, we cannot exclude that the detected changes might also (partly) track progression of other neurodegenerative diseases. Thus, before putative diagnostic application, this needs to be further assessed. However, this does not reduce the potential differentiating significance of this biomarker panel for prognostic application in a known (pre)symptomatic HD carrier setting.

In conclusion, we describe the development of a panel of candidate HD biomarkers that can be easily measured by transcript analysis of whole blood and that may have application in disease staging and the monitoring of therapeutic effectiveness. Longitudinal and cross-sectional studies in additional cohorts will be needed to further validate this panel before its application in the clinic. Finally, the assesment of the disease relevance of the genes involved may well contribute to finding new HD therapeutic targets.

## Figures and Tables

**Table 1:** DeepSAGE top 10 upregulated (coefficient +) and downregulated (coefficient −) genes in HD blood samples

*Gene*	*Description*	*Coefficient*^a^	*Expression*^b^	*Adjusted P-Value*	*Protein Function*
HYAL2	Hyaluronoglucosaminidase 2	+0.4	2.6	1.0E−03	Hydrolyzes hyaluronic acid
LMO2	LIM domain only 2	+0.3	6.6	1.0E−03	Yolk sac hematopoiesis
MARC1	Mitochondrial amidoxime reducing C1	+0.4	5.0	5.0E−03	N-hydroxylate prodrug conversion
NT5DC2	5′-Nucleotidase domain containing 2	+0.4	2.8	9.0E−03	Hydrolase and metal ion binding
RNF135	Ring finger protein 135	+0.3	5.8	9.0E−03	DDX58 Ubiquitination~IFN-β
PROK2	Prokineticin 2	+0.5	7.9	1.0E−02	Circadian clock—GI contraction
RPN1	Ribophorin I	+0.3	5.5	1.0E−02	26S Proteasome ubiquitin binding
CYSTM1	Cysteine-rich transmembrane module 1	+0.4	6.0	1.0E−02	Stress tolerance
VCAN	Versican	+0.3	8.2	1.6E−02	Intercellular signaling Binds hyal. acid
NCF4	Neutrophil cytosolic factor 4	+0.3	8.9	1.8E−02	NADPH-oxidase component
ARL4C	ADP-Ribosylation factor-like 4C	−0.3	8.2	1.0E−03	Microtubule vesicular transport
TMEM109	Transmembrane protein 109 (Mg23)	−0.3	7.0	6.0E−03	UVC *α*B-Crystallin protection
MACF1	Microtubule-actin crosslinking factor 1	−0.2	7.2	6.0E−03	Actin-microtubule stabilization
MDN1	Midasin homolog	−0.2	5.3	7.0E−03	AAA-ATPase(dynein)
PTPN4	Protein tyrosine phosphatase NR type 4	−0.3	5.1	9.0E−03	Glutamate receptor signaling
PRF1	Perforin 1	−0.4	9.5	1.0E−02	Cytolysis
CD3G	CD3g Molecule gamma	−0.3	7.5	1.0E−02	CD3 complex signal transduction
NMT2	N-Myristoyltransferase 2	−0.3	3.4	1.0E−02	N-terminal Myristoylation
KLRD1	Killer cell lectin receptor subfamily D 1	−0.4	6.1	1.0E−02	Recognition of MHC class I HLA-E
GPR56	G Protein-coupled receptor 56	−0.4	7.3	1.0E−02	Brain cortical patterning

a Coefficients of gene expression change per motor score unit multiplied by average motor score.

b Average log_2_ gene expression levels. Protein function based on Genecards.

**Table 2:** Fluidigm RT-qPCR technical and biological validation results of DeepSAGE genes

		*Discovery cohort (*n*=25)*		*Independent cohort (*n*=23)*		*All samples (*n*=48)*
*Gene*	*Description*	P*-value*	*Coeff.*^a^	P*-value*	*Coeff.*	P*-value*
CYSTM1	Cysteine-rich transmembrane module 1	6.0E−03	0.5	2.0E−03	0.5	1.0E−04
PROK2	Prokineticin 2	1.0E−02	1.0	2.0E−03	0.7	1.0E−03
AQP9	Aquaporin 9	2.0E−03	0.5	6.0E−05	0.7	2.0E−05
ZNF238	Zinc finger protein 238	2.0E−02	0.3	8.0E−03	0.5	1.0E−03
ANXA3	Annexin 3	4.0E−02	0.5	6.0E−03	0.7	7.0E−04
RNF135	Ring finger protein 135	7.0E−02	0.2	7.0E−03	0.2	6.0E−03
LMO2	LIM domain only 2	3.0E−02	0.2	7.0E−02	0.2	6.0E−03
ARL4C	ADP-ribosylation factor like 4	3.0E−02	−0.3	9.0E−01	0.02	7.0E−03
TMEM109	Transmembrane protein 109	4.0E−02	−0.2	5.0E−01	−0.04	1.0E−02
CEBPA	CCAAT/enhancer binding A	2.0E−02	0.4	1.0E−01	0.2	1.0E−02
MACF1	Microtubule-actin crosslinking F1	2.0E−02	−0.3	6.0E−01	−0.04	1.0E−02
PTPN4	Protein tyrosine phosphatase NR 4	1.0E−02	−0.35	6.0E−01	−0.04	3.0E−02
MARC1	Mitochondrial amidoxime reducing C1	2.0E−02	0.5	1.0E−01	0.4	6.0E−02
H2AFY	H2A histone family, member Y	1.0E−01	0.15	1.0E−01	0.15	1.0E−01
HYAL2	Hyaluronoglucosaminidase 2	1.0E−01	0.20	4.0E−02	0.2	1.0E−01
NOL3	Nucleolar protein 3	2.0E−01	0.15	8.0E−02	0.2	2.0E−01
MDN1	Midasin homolog	2.0E−01	−0.15	9.0E−01	NC^b^	1.0E−01
NT5DC2	5′-Nucleotidase domain containing 2	5.0E−01	0.1	2.0E−01	0.4	3.0E−01
RGS14	Regulator of G-protein signaling 14	1.0E−01	0.2	5.0E−01	0.2	3.0E−01
TAF15	TATA box—associated factor	1.0E−01	−0.1	2.0E−01	0.15	2.0E−01

a Coeff.=Coefficients of gene expression change per motor score unit multiplied by group average motor score.

b NC=No change.
