# Supplementary figures and images for: Huntington's disease biomarker progression profile identified by transcriptome sequencing in peripheral blood
Source: Eur J Hum Genet. 2015 Jan 28;23(10):1349–56. doi: 10.1038/ejhg.2014.281 (PMC4592077; doi:10.1038/ejhg.2014.281)

Supplementary Figure S5

A

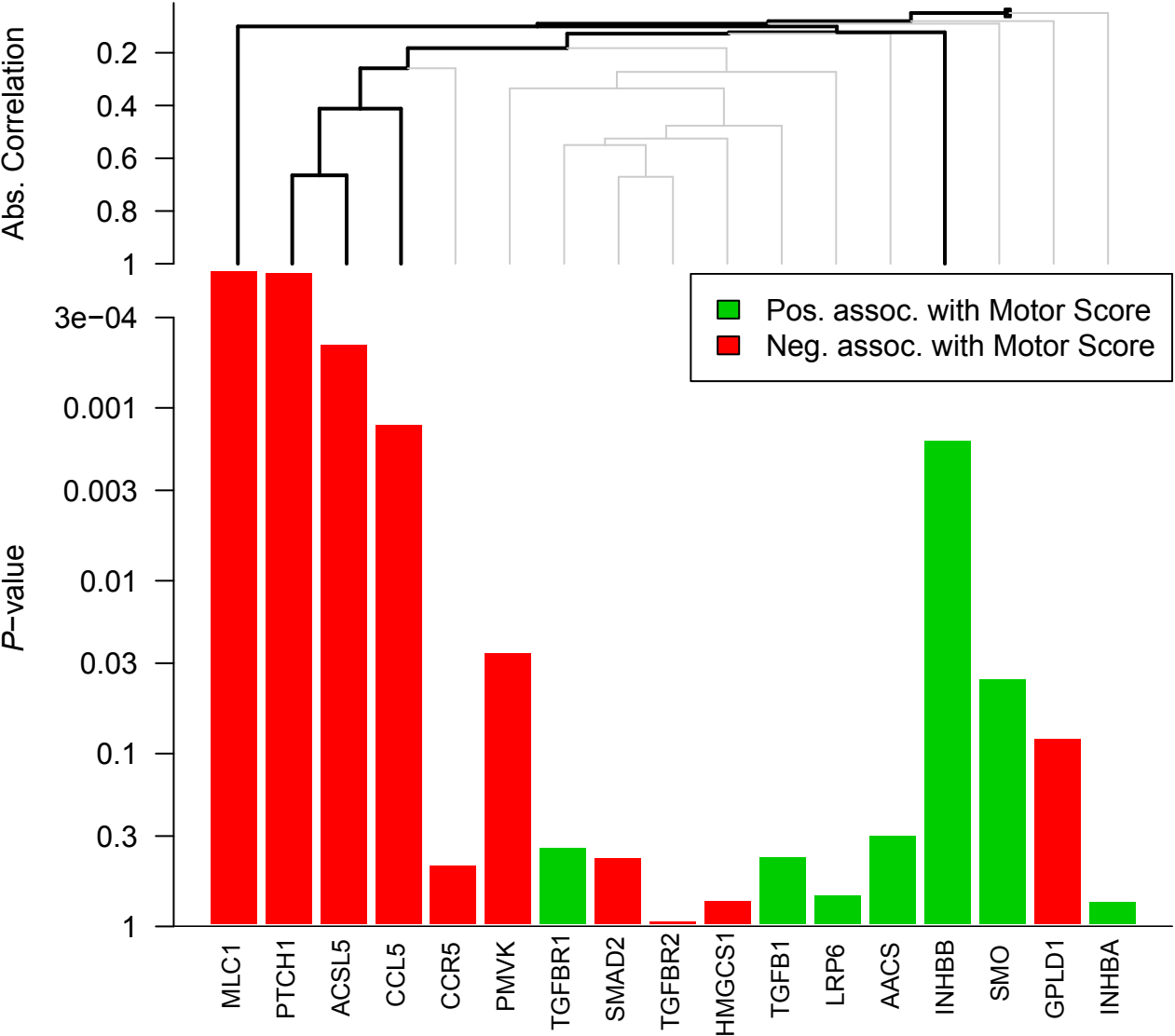

B

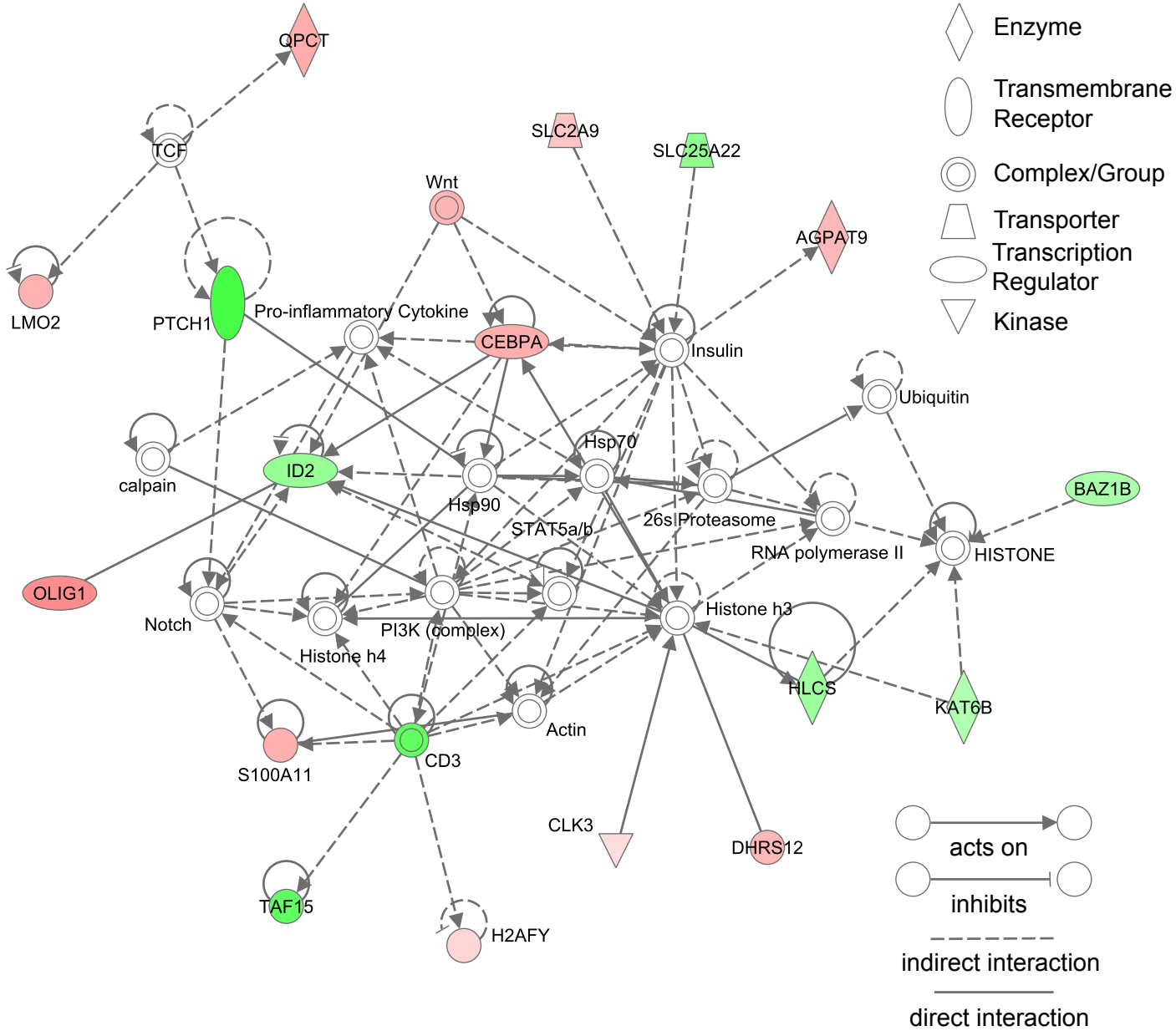

Supplement: Supplementary Figure S5 [file ejhg2014281x1.pdf]
