# Supplementary material for: Huntington's disease biomarker progression profile identified by transcriptome sequencing in peripheral blood
Source: Eur J Hum Genet. 2015 Jan 28;23(10):1349–56. doi: 10.1038/ejhg.2014.281 (PMC4592077; doi:10.1038/ejhg.2014.281)
Supplement: Supplementary Figure S5 Legend [file ejhg2014281x2.docx]

**Titles and legends to figures**

**Supplementary Figure S5** A. Covariate plot analysis from global test for the GO-term “response to cholesterol”. All genes found in the DeepSAGE analysis and for the above term are shown on the x axis, while the P-values for these individual genes are plotted on the y-axis (most significant are represented by highest bars, red are negatively associated with TMS, green are positively associated with TMS). The dendrogram reflects the correlation in expression of these genes across all samples, with bold lines indicating branches (groups of genes) that are significant B. Molecules reported from IPA top diseases and functions network analysis and for the terms skeletal and muscular disorders, connective tissue disorders and cancer (colored genes represent genes discovered from DeepSAGE sequencing, red=upregulated genes, green=downregulated genes).
