# Supplementary material for: Huntington's disease biomarker progression profile identified by transcriptome sequencing in peripheral blood
Source: Eur J Hum Genet. 2015 Jan 28;23(10):1349–56. doi: 10.1038/ejhg.2014.281 (PMC4592077; doi:10.1038/ejhg.2014.281)
Supplement: Supplementary Table S3 [file ejhg2014281x3.docx]

**Supplementary Table S3** Cohort characteristics of samples used for DeepSAGE gene expression profiling and Fluidigm RT-qPCR validation analysis.

| **Groups** | **HD state** | **Gender** | **n** | **Age Mean (Range)** | **TMS^a^ Mean (Range)** | **TFC^b^ Mean (Range)** |
| --- | --- | --- | --- | --- | --- | --- |
| DeepSAGE  Primary  Cohort  n=124 | Controls  n=33 | Female | 18 | 44 (25-68) | 1.9 (0-11) | 12.7 (11-13) |
|  |  | Male | 15 | 39 (23-58) | 2 (0-12) | 12.9 (12-13) |
|  | Presymptomatics  n=27 | Female | 17 | 41 (26-57) | 2.6 (0-5) | 11.7 (5-13) |
|  |  | Male | 10 | 43 (31-55) | 2.1 (0-5) | 12.8 (11-13) |
|  | Symptomatics  n=64 | Female | 32 | 52 (22-76) | 40.5 (7-107) | 5.9 (0-13) |
|  |  | Male | 32 | 55 (32-74) | 34.2 (7-86) | 6.9 (0-13) |
| Fluidigm  Discovery  Cohort  n=25 | Controls  n=11 | Female | 6 | 46 (25-68) | 3.3 (0-11) | 12.5 (11-13) |
|  |  | Male | 5 | 42 (35-55) | 2.2 (0-8) | 12.8 (12-13) |
|  | Carriers  n=14 | Female | 6 | 46 (22-54) | 46.5 (13-82) | 5.8 (2-12) |
|  |  | Male | 8 | 62 (37-73) | 53.6 (16-86) | 4.9 (0-13) |
| Fluidigm  Independent  Cohort  n=23 | Controls  n=12 | Female | 6 | 46 (38-55) | 1.8 (0-4) | 13 |
|  |  | Male | 6 | 48 (35-58) | 2.3 (0-6) | 13 |
|  | Carriers  n=11 | Female | 7 | 52 (33-61) | 26.6 (10-45) | 9.9 (7-13) |
|  |  | Male | 4 | 52 (40-61) | 22.0 (10-41) | 10.3 (7-13) |

^a^ Total Motor Score (0-124) of the Unified Huntington’s Disease Rating Scale, with higher scores indicating more motor symptoms.^b^ Total Functional Capacity of the Unified Huntington’s Disease Rating Scale, with lower scores indicating worse functional capacity
