# Supplementary material for: Huntington's disease biomarker progression profile identified by transcriptome sequencing in peripheral blood
Source: Eur J Hum Genet. 2015 Jan 28;23(10):1349–56. doi: 10.1038/ejhg.2014.281 (PMC4592077; doi:10.1038/ejhg.2014.281)
Supplement: Supplementary Table S4 [file ejhg2014281x4.docx]

**Supplementary Table S4** Sample RNA and sequencing quality characteristics for all 124 samples used for DeepSAGE gene expression profiling.

| **Average RNA Quality**  **(Range 1-10)** | 8.4  (7 - 9.6) |
| --- | --- |
| **Average Number of**  **Total Reads Sequenced** | 23.5 Million  (11 – 37) |
| **Average Percentage of Total Reads Aligned ^a^** | 87.1% (20.4 M)  (73 – 97 %) |
| **Average Percentage of Total Reads Supressed ^b^** | 9.37 % (2.2 M)  (2 – 17 %) |
| **Average Percentage of Total Reads Failed To Align** | 3.54 % (0.8 M)  (0.6 - 15 %) |

^a^ At least one reported alignment.

^b^ Bowtie –m option - suppress all alignments for a particular read or pair if more than 2 reportable alignments exist for it.
