# Supplementary material for: Huntington's disease biomarker progression profile identified by transcriptome sequencing in peripheral blood
Source: Eur J Hum Genet. 2015 Jan 28;23(10):1349–56. doi: 10.1038/ejhg.2014.281 (PMC4592077; doi:10.1038/ejhg.2014.281)
Supplement: Supplementary Table S7 [file ejhg2014281x7.docx]

**Supplementary Table S7** Global test pathway analysis of DeepSAGE gene expression data for KEGG pathways, GO terms and MicroRNA target sets.

| **KEGG Pathways** | **Genes** | **GO Terms** | **Genes** | **Micro-RNAs** | **Genes** |
| --- | --- | --- | --- | --- | --- |
| Toll-like receptor signaling | 82 | NADP binding | 35 | MiR-504 | 71 |
| Pentose phosphate | 22 | Striated muscle cell differentiation | 119 | MiR-138 | 191 |
| Osteoclast differentiation | 115 | Lung alveolus development | 25 | MiR-337 | 128 |
| Jak-STAT signaling | 102 | Pancreas development | 42 | MiR-218 | 305 |
| Amyotrophic lateral sclerosis | 39 | Cellular response to light stimulus | 28 | MiR-522 | 138 |
| Neuroactive ligand receptor interaction | 97 | Response to cholesterol | 17 | MiR-492 | 51 |
| Pathways in cancer | 266 | Positive regulation of IL-6 production | 25 | MiR-520D | 273 |
| Type II diabetes mellitus | 36 | Negative regulation of IL-12 production | 10 | MiR-498 | 92 |
| Long-term depression | 50 | Cellular carbohydrate biosynthetic process | 52 | MiR-28 | 84 |
| Chagas disease | 90 | Glucose catabolic process | 62 | MiR-129 | 151 |
| Focal adhesion | 154 | Regulation of heart contraction | 54 | MiR-513 | 104 |
| Cytokine-cytokine receptor interaction | 171 | Positive regulation of neurological system | 26 | MiR-18A,MiR-18B | 104 |
