# Supplementary material for: Huntington's disease biomarker progression profile identified by transcriptome sequencing in peripheral blood
Source: Eur J Hum Genet. 2015 Jan 28;23(10):1349–56. doi: 10.1038/ejhg.2014.281 (PMC4592077; doi:10.1038/ejhg.2014.281)
Supplement: Supplementary Table S8 [file ejhg2014281x8.docx]

**Supplementary Table S8** Ingenuity pathway analysis top disease and functions for the top DeepSAGE differentially expressed genes.

| **ID** | **Top Diseases and Functions** | **Score** | **Focus Molecules** |
| --- | --- | --- | --- |
| 1 | Cell Death and Survival, Nervous System Development and Function, Tissue Development | 33 | 21 |
| 2 | Cell-To-Cell Signaling and Interaction, Hematological System Development, Immune Cell Trafficking | 30 | 21 |
| 3 | Hematological System Development and Function, Inflammatory Response, Tissue Morphology | 29 | 18 |
| 4 | Infectious Disease, Renal and Urological Disease, Lipid Metabolism | 28 | 18 |
| 5 | Cell Cycle, Cell Morphology, Cell-To-Cell Signaling and Interaction | 27 | 17 |
| 6 | Cancer, Connective Tissue Disorders, Skeletal and Muscular Disorders | 26 | 17 |
| 7 | Carbohydrate Metabolism, Small Molecule Biochemistry, Immunological Disease | 25 | 16 |
| 8 | Hereditary Disorder, Renal and Urological Disease, Carbohydrate Metabolism | 24 | 16 |
| 9 | Cell Cycle, Skeletal and Muscular System Development and Function, Cell Death and Survival | 24 | 16 |
| 10 | Auditory Disease, Immunological Disease, Cellular Movement | 21 | 14 |
